# Mosquito-borne viral diseases in the Democratic Republic of the Congo: a review

**DOI:** 10.1186/s13071-020-3985-7

**Published:** 2020-02-27

**Authors:** Kennedy M. Mbanzulu, Leonard E. G. Mboera, Flory K. Luzolo, Roger Wumba, Gerald Misinzo, Sharadhuli I. Kimera

**Affiliations:** 1 SACIDS-Africa Centre of Excellence for Infectious Diseases of Humans and Animals in Eastern and Southern Africa, Sokoine University of Agriculture, P.O. Box 3297, Chuo Kikuu, Morogoro, Tanzania; 2 Department of Tropical Medicine, Infectious and Parasitic Diseases, University of Kinshasa, P.O. Box 747, Kinshasa, Democratic Republic of the Congo; 3 Department of Veterinary Microbiology, Parasitology and Biotechnology, Sokoine University of Agriculture, P.O. Box 3019, Chuo Kikuu, Morogoro, Tanzania; 4 Department of Veterinary Medicine and Public Health, Sokoine University of Agriculture, P.O. Box 3021, Chuo Kikuu, Morogoro, Tanzania

**Keywords:** Mosquito-borne viruses, Epidemiology, Ecology, Democratic Republic of the Congo

## Abstract

**Background:**

Mosquito-borne viral infections have in recent years, become a public health threat globally. This review aimed to provide an overview of the ecological and epidemiological profiles of mosquito-borne viral infections in the Democratic Republic of the Congo (DRC).

**Methods:**

A search of literature was conducted using Google Scholar, PubMed and the WHO website using the following keywords: “Democratic Republic of the Congo”, “Zaire”, “Belgian Congo” and either of the following: “mosquito-borne virus”, “arbovirus”, “yellow fever”, “dengue”, “chikungunya”, “West Nile”, “Rift Valley fever”, “O’nyong’nyong”, “Zika”, “epidemiology”, “ecology”, “morbidity”, “mortality”. Published articles in English or French covering a period between 1912 and October 2018 were reviewed.

**Results:**

A total of 37 articles were included in the review. The findings indicate that the burden of mosquito-borne viral infections in DRC is increasing over time and space. The north-western, north-eastern, western and central regions have the highest burden of mosquito-borne viral infections compared to south and eastern highland regions. Yellow fever, chikungunya, dengue, Zika, Rift Valley fever, West Nile and O’nyong’nyong have been reported in the country. These mosquito-borne viruses were found circulating in human, wildlife and domestic animals. Yellow fever and chikungunya outbreaks have been frequently reported. *Aedes aegypti* and *Ae. simpsoni* were documented as the main vectors of most of the mosquito-borne viral infections. Heavy rains, human movements, forest encroachment and deforestation were identified as drivers of mosquito-borne viruses occurrence in DRC.

**Conclusions:**

Mosquito-borne viral infections are becoming common and a serious public health problem in DRC. In the current context of climate change, there is urgent need to improve understanding on ecological and epidemiology of the diseases and strengthen surveillance systems for prompt response to epidemics in DRC.

## Background

Arthropod-borne viruses are referred to as viruses transmitted either to animals or humans by blood-sucking arthropods [1]. There are over 700 known arthropod-borne viruses and at least 80 immunologically distinct types that cause diseases in humans [2]. Most of the arthropod-borne viral infections are transmitted by mosquitoes. Transmission of these viruses to humans involves complex processes influenced by the mosquito and viral genetics, environmental factors and human activities [3]. Mosquitoes belonging to the genus *Aedes* are the major vectors of Chikungunya (CHIKV), Dengue (DENV), Rift Valley fever (RVFV), Yellow fever (YFV) and Zika (ZIKV) [4]. These mosquito-borne viral diseases are often misdiagnosed with other febrile illnesses, such as malaria due to lack of differential diagnostic tools [5]. The geographical distribution patterns of these mosquito-borne viruses are related to the presence of their mosquito vectors; and overlap as they share common vectors. Therefore, co-infection or co-occurrence of more than one mosquito-borne virus species in a certain geographical location is common [6].

The Democratic Republic of the Congo (DRC) is located in central Africa. It is the second largest and one of the most populated countries in Africa. The DRC harbours rich fauna, flora and biodiversity which offer suitable opportunity for the emergence of numerous viral diseases. A number of mosquito-borne virus species have been documented in some regions of DRC during the last century. The first yellow fever outbreak in DRC was reported from Kongo-Central in 1912 [7]. Thereafter a number of studies on YFV and other mosquito-borne viral infections were carried out and extended to other geographical locations until the end of colonial period in 1960 [8]. During the past two decades, repeated outbreaks of Chikungunya and Yellow fever have been reported in DRC. During the same time, the circulation of other mosquito-borne viruses of public health importance such as DENV, ZIKV, RVFV and West Nile virus (WNV) have also been recorded [9–11]. This review attempts to summarize the available data on mosquito-borne viral infections in DRC in order to provide an insight of their geographical distribution, virus species diversity, mosquito vectors and animal reservoirs. In addition, the review explores the risks factors associated with their transmission and epidemic occurrence.

## Methods

### Search strategy

**Fig. 1:**
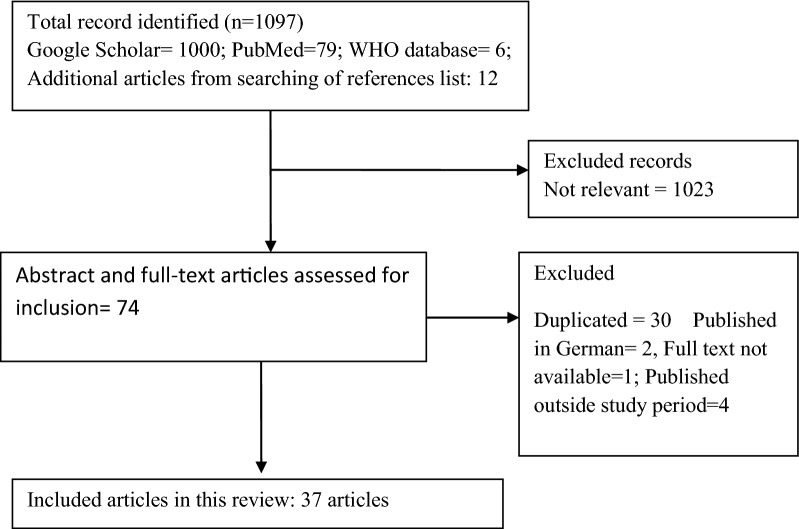
PRISMA flow chart diagram describing the studies selection process

Using PRISMA guidelines for systematic review, a literature search was carried out using Google Scholar and PubMed databases to find relevant information related to ecology and epidemiology of mosquito-borne viral infections in the DRC. Combinations of search keywords were used (“Belgian Congo” OR “Democratic Republic of the Congo” OR “Zaïre”) AND (“mosquito-borne virus” OR arbovirus OR “arthropod-borne virus” OR “yellow fever” OR “chikungunya” OR “dengue” OR “Rift Valley Fever virus” OR “West Nile virus” OR “Zika” OR “alphavirus” OR “flavivirus” OR “bunyavirus”). Additional information was searched from the World Health Organization (WHO) (http://www.who.int) database. Articles in either English or French were included in the review. Initially, titles and abstracts were screened, then full text of articles identified as possibly relevant were reviewed. The bibliographies of included articles were assessed for further relevant publications. In case of duplicated articles, only one was considered in this study (Fig. 1).

### Eligibility and inclusion criteria

The focus was on the articles that described mosquito-borne viruses in DRC between 1912 and October 2018. The information extracted from the reviewed articles included studies on the epidemiology and ecology of arboviruses including geographical location, study population, diagnostic method used, virus species detected, serotypes and genetic diversity, number of cases or prevalence, risk factors of transmission, mosquito vector and their habitats, potential host or reservoir, seasonality, epidemics/outbreaks, mortality and control options.

### Exclusion criteria

Abstracts without available full text, articles in language other than English or French, review article duplicated information, studies describing only diagnostic methods or vaccine development were excluded.

### Analysis

The results were described using figures to show the trends of distribution of mosquito-borne viruses occurrence over time and space. To map geographical distribution of mosquito-borne viruses in the DRC, geographical coordinates of study areas recorded in the present review were searched in the database of the Central African Satellite Forest Observatory (OSFAC), Kinshasa, DRC and associated with the respective mosquito-borne virus presence recoded. It was possible to obtain some precise details regarding the geographical areas of the reviewed articles by emailing authors directly. The maps were drawn using QGIS 2.2.0. (QGIS Development team, GNU General Public License).

## Results

**Fig. 2:**
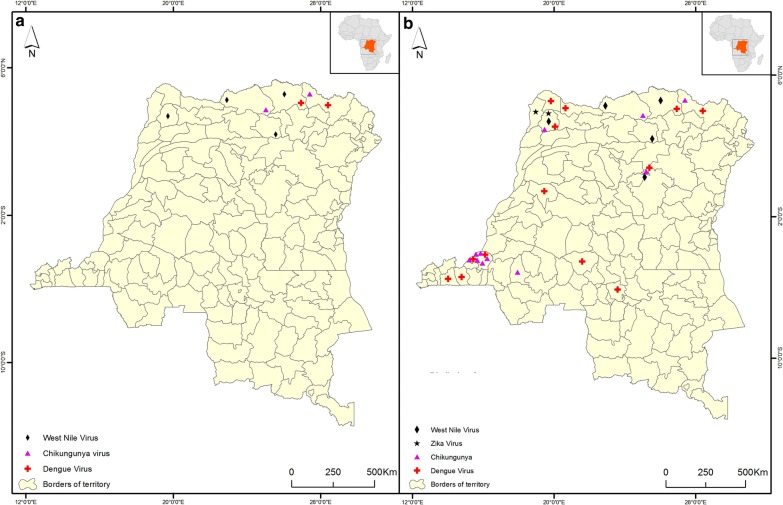
Distribution of mosquito-borne virus occurrence in different territories in the DRC, 1917–2018. **a** Distribution of mosquito-borne virus occurrence before 1998. **b** Distribution of mosquito-borne virus occurrence in between 1998 and 2018. *Key*: black rhombus, West Nile virus; purple triangle, chikungunya virus; red cross, dengue virus; black star, Zika virus; black line, borders of territories

A total of 69 articles were retrieved and 37 articles covering a period between 1912 and October 2018 were included in the present review. Seven mosquito-borne viruses belonging to three families, the *Flaviviridae*, *Bunyaviridae* and *Alphaviridae*, were recorded across the DRC. Among the *Flaviviridae*, four species, YFV, DENV, ZIKV and WNV, were recorded. Among the *Alphaviridae*, two species, CHIKV and ONNV were reported. The recorded mosquito-borne viruses occurred either during an outbreak investigation, immediately after an outbreak, or occasionally during inter-epidemic periods. Most of the records were related to YFV and CHIKV. A number of studies were carried during colonial era before 1960 and around the beginning of the country’s independence in 1960. Most of the reports on mosquito-borne diseases were published between 1998 and 2018 (Fig. 2). Only two recent entomological studies were recorded in DRC, one study on *Aedes* fauna in Kinshasa [12] and another one on mosquito virus infectivity [13].

These viruses were identified using different diagnostic methods ranging from serological investigation [animal inoculation, enzyme‐linked immunosorbent assay (ELISA), western blot, plaque reduction neutralization test (PRNT)], morphological investigation of affected organs, tissue culture cell lines (Vero, C6/36) and molecular biological methods including reverse transcription polymerase chain reaction (RT-PCR) detection, genome sequencing and molecular genetics analysis. The majority of sample specimens analysed were from humans; few were from animals and mosquitoes. Some studies reported co-occurrence or co-infections of mosquito-borne viruses. These included CHIKV and YFV, CHIKV and DENV, YFV and DENV [9, 10, 14–16].

### Yellow fever virus

The first yellow fever outbreak in DRC was reported among ship passengers from Europe in 1912 at Matadi Beach [7]. Later in 1928, another YFV outbreak occurred in the same area. This was the first autochthonous YFV outbreak in the DRC and it caused a mortality rate as high as 63.4% [17]. From 1948 to 1957, yellow fever cases were regularly recorded in DRC. In 1958, the country experienced two major outbreaks: first in Gemena township of the Sud-Ubangi Province (case fatality rate of 36.5%) [18] and another outbreak (co-infection with chikungunya) in the Territory of Dungu from Haut-Uelé Province [14]. Afterwards, in 1960, another yellow fever outbreak was reported from Bili village in Bondo territory of Bas-Uélé Province [15]. After the independence in 1960, yellow fever outbreaks kept on recurring in all 26 provinces of DRC. From 2001 to 2004, a nationwide surveillance reported about 400 yellow fever suspected cases annually without a biological confirmation and a lethality rate of 3% in 2004 [10]. In a demographic survey of 2013–2014, an overall YFV prevalence of 31.5% among children was reported in DRC [9]. Despite the implementation of a yellow fever vaccination programme (involving children 9 months-old) in DRC since 2003, most vaccinated children failed to demonstrate sufficient proof of seroconversion [9]. From 2010–2016, four yellow fever outbreaks resulting from either autochthonous or imported infections were reported in DRC [19–22]. The 2016 outbreak was the largest with causing a total of 393 deaths. While, initially the majority of recorded yellow fever cases were imported from neighbouring Angola, 90% of deaths in 2016 were recorded in a single Province of Kongo-Centrale [23]. The autochthonous cases of YFV have also been documented in the provinces of Bas-Uele, Equateur, Kasai-Central, Kinshasa, Kwango, Lualaba, Tshuapa and Kongo-Central [22] (Table 1).

**Table 1:** Reported occurrence of yellow fever in DRC, 1912–2018

Province (specific area)	No. of cases	No. of deaths	Period of record	References
Kongo-central (Matadi)	6		1917	[7]
Kongo-central (Matadi, Boma)	41	26	1928	[17]
Haut-Uelé (Dungu)			1958	[14]
Sud-Ubangi (Gemena)	60	23	1958	[18]
Bas-Uélé (Bili village from Bondo)			1961	[15]
Bas-Uélé (Titule Health Zone)	11	2	2010	[19]
Lomami (Lubao Health Zone) Kasai-Orientale (KamanaLudimbi-Lukula Health Zone)	51	19	2013	[20]
Bas-Uélé (Bondo, Buta), Haut-Lomami (Kikondja)	139	6	2014	[21]
Kinshasa, Kongo-central, Bas-Uele, Equateur, Kasai-central, Kwango, Lualaba, Tshuapa	2987	121	2016	[22]
Kongo-central (Nsonapangu, Kimpese, Kimpangu, Matadi, Moanda, Boma, Kitona, Masa)	393	42	2016	[23]

**Fig. 3:**
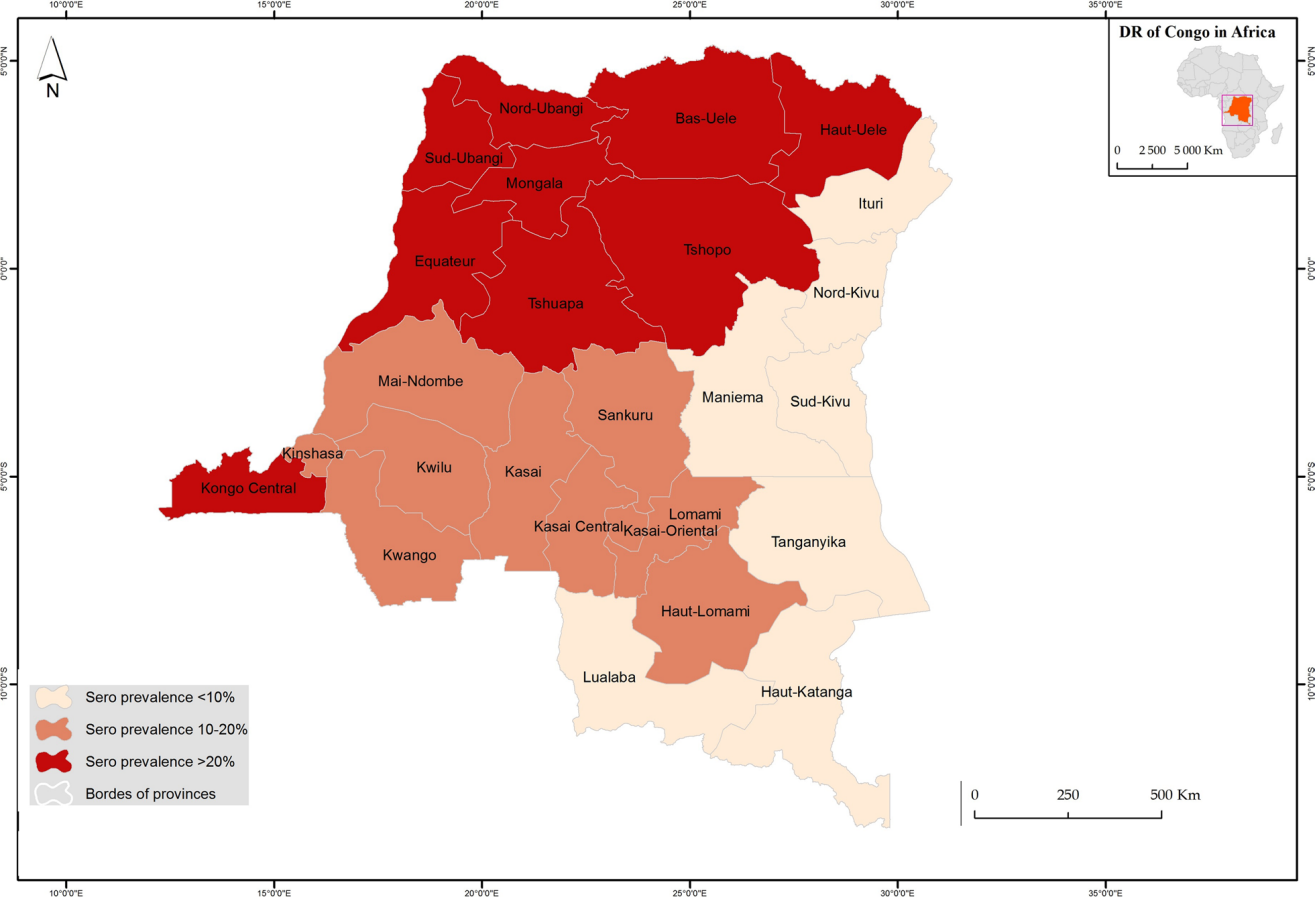
Geographical distribution of yellow fever virus seroprevalence in different provinces of the DRC, 1917–2018. *Key*: light pink, provinces with YFV seroprevalence < 10%; dark pink: provinces with YFV seroprevalence ranging between 10–20%; red, provinces with YFV seroprevalence > 20%; white line: borders of provinces

The Democratic Republic of the Congo belongs to an endemic geographical area at high risk for YFV transmission [24]. YFV transmission in DRC varies from one region to another and within the region. The absence of YFV circulation or lowest seroprevalence (< 5%) has been observed from the provinces located in high altitudes covering the whole eastern region (Ituri, Sud-Kivu, Nord-Kivu, Maniema) and the southern region from former Katanga province (currently divided into Tanganyika, Haut-Lomami, Haut-Katanga, Lualaba) [25]. The highest seroprevalence of YFV (≥ 30%) has been recorded from the savannah regions intersected by forest galleries from northern regions and the Atlantic coastal region in western part of DRC [25, 26]. The identified territories with high YFV transmission were Bosobolo and Mobayi-mbongo in the Province of Nord-Ubangi in the North-West, Ango and Bondo in the Province Bas-Uélé in the North-East region and in the Province of Kongo-Central in western DRC [25]. Lower seroprevalence rates (10–20%) have been reported along the Equator forest and in the central part of the country (Mai-Ndombe, Equateur, Kasai, Kasai-Central, Kasai-Oriental and Lomami provinces), as well as from provinces of Kwilu and Kwango in the western region [27] (Fig. 3).

### Chikungunya

The two first reported chikungunya cases in the DRC occurred during a yellow fever outbreak in north-eastern region. The first was in 1958 in Haut-Uelé Province [14], and the second in 1960 in the neighbouring Province of Bas-Uélé in Bondo territory [15]. Since then, no circulation of CHIKV was documented in DRC until 1998 [11]. In 1998, of 45 patients sampled during an outbreak of WNV in Kisangani City in Tshopo Province of the north-eastern region, 28.8% patients were found positive for CHIKV immunoglobulin M (IgM) antibodies [11]. Kinshasa, the capital of DRC experienced three chikungunya outbreaks during 1999 and 2000, with an estimated 50,000 suspected cases [28]. These outbreaks occurred after heavy rains in the urban areas of Matete and Kingabwa municipalities. The third occurred in the sub-rural area of Ndaku-ya-Mpembe in the Kinkole municipality. More than half (57.9%) the samples tested positive for CHIKV IgM antibodies and nine strains were isolated in C6/36 cells [28]. Furthermore, another outbreak was recorded in 2012 from three new areas (Maluku, Mont-ngafula and Mbinza Meteo health zones) in Kinshasa [16]. Of 10 cases of chikungunya recorded among Belgian travellers between 2007 and 2012, six were from DRC [29]. In a retrospective countrywide surveillance study carried out between 2003 and 2011, of the 453 sera screened, two positive cases of CHIKV were detected by RT-PCR. One case was from Gemena in the Sud-Ubangi Province in north-western DRC and the other from the Province of Kwilu [10]. In a survey carried out in 2015, from 96 patients tested negative for malaria in Kinshasa, the seroprevalence of CHIKV was 9.6% and 5 samples were found positive for CHIKV by RT-PCR [30]. Based on a genetic study, the isolates of CHIKV that were found to circulate in DRC belonged to the Congo genotype which is closely related to the Central African Republic and Uganda isolates [31] (Table 2). African buffalo, chimpanzee and elephant from Haut-Uelé Province have been identified as potential hosts or reservoirs of CHIKV by detecting the presence of antibodies from these species (Table 3) [32, 33].

**Table 2:** Recorded studies on chikungunya in DRC, 1958–2018

Province (specific area)	Laboratory test	No. of samples tested (% positive)	Period of record	References
Haut-Uelé (Dungu in Doruma)	Animal inoculation	3 (100)	October-December 1958	[14]
Bas-Uelé (Bondo in Bili)	Animal inoculation	36 (44.0)	Rainy season of 1960	[15]
Tshopo (Kisangani)	ELISA (IgM)	45 (28.8)	February-March 1998	[11]
Kinshasa: Matete (Des Marais), Kingabwa (Bribano), Kinkole (NdakuyaPembe)	ELISA IgM, Cell C6/36	76 (57.8)	February-May 1999; May-July 2000	[28]
Whole country	RT-PCR	453 (0.4)	2003–2012	[10]
Sud-Ubangi (Gemena), Kwilu	RT-PCR	110 (1.8))	2003–2012	[10]
DRC/Belgium	RT-PCR, ELISA		2012	[29]
Kinshasa (Binza-Meteo, Mont-Ngafula, Maluku)	ELISA		2012	[16]
Kinshasa	RT-PCR, ELISA	10 (9.6) IgM; 5 (4.8) in RT-PCR	2014	[30]

**Table 3:** Reported mosquito-borne viruses circulating in diverse animals and mosquitoes in DRC, 1960–2018

Animal/mosquito	CHIKV	DENV	YFV	RVFV	WNV	ONNV	Alphavirus	Flavivirus	Study location
Chimpanzee	+	+	+		+				Haut-uelé (Ango, Bondo) [32]
Elephant	+				+		+	+	Garamba National Park [33]
Buffalo	+	+			+	+	+	+	Garamba National Park [33]
Duiker						+	+	+	Ituri Rain Forest [33]
Gorillas							+	+	Virunga, Garamba National Park [33]
Cattle				+					Katanga, Nord Kivu (Beni, Lubero, Butembo, Masisi, Rutshuru) Sud Kivu (Kabare, Mwenga, Shabunda) Ituri (Aru, Mahagi) [34, 35]
Dog					+				Kinshasa [39]
Horse					+				Kinshasa [38]
*Aedes* spp.	+			+			+	+	Kinshasa [13]
*Anopheles* spp.						+			Kinshasa [13]
*Culex* spp.								+	Kinshasa [13]

*Key*: +, screened positive for virus

### Dengue

Three serotypes of dengue virus (DENV-1, DENV-2 and DENV-3) have been recorded in DRC, the most frequent being serotype DENV-1 [9, 10, 30]. The first case of dengue was reported from Ango and Bongo territories from the Province of Haut-Uelé, where a dengue seroprevalence of 68.8% was reported in 1960 [32]. The prevalence in four studies carried out between 1998 and 2015 using different diagnostics methods (ELISA, PRNT, rapid diagnostic test and RT-PCR) ranged from 2.7% to 8.8% [9, 11, 30]. In Kisangani of Tshopo Province, DENV was reported to co-circulate with CHIKV during the West Nile outbreak of 1998 [11]. In Kinshasa, co-occurrence of dengue and chikungunya was reported during the outbreak of 2012 [16]. Dengue occurrence has been reported from western (Kinshasa, Kongo central), central (provinces of Kasai, Kasai Oriental), north-western (Equateur, Mongala, Sud Ubangi, Nord Ubangi) and north-eastern regions (Tshopo, Haut-uélé, Bas-Uélé) (Table 4). Antibodies against DENV have also been reported from chimpanzee in 1960 and from buffalo in 2013 [32, 33].

**Table 4:** Reports on dengue in DRC, 1960–2018

Province (specific areas)	Laboratory test	No. of samples tested (% positive)	Period of record	References
Haut-Uelé, Bas-Uéle (Ango-Bondo)	Animal inoculation	32 (68.7); DENV-2 (95.0); DENV-1 (5.0)	October–December 1960	[32]
Tshopo (Kisangani)	ELISA (IgM)	45 (8.8)	March 1998	[11]
Whole country	RT-PCR	453 (3.5); DENV-1 (85.0); DENV-2 (15.0)	2003–2012	[10]
Equateur (Health Zone Ingende), Sud-Ubangi (Gemena), Nord Ubangi (Health Zone Mobayi-mbongo, HZ Bili from Bosobolo)	RT-PCR	110 (3.6)	2003–2012	[10]
Bas-Uelé (Health Zones Titule, Lubunga) Tshopo (Makiso-Kisangani, Lubinga)	RT-PCR	168 (4.7)	2003–2012	[10]
Kasai Oriental (Health Zone Diulu in Mbujimayi)	RT-PCR	51 (1.9)	2003–2012	[10]
Kasai (Health Zone Kakenge from Mweka)	RT-PCR	41 (2.4)	2003–2012	[10]
Kongo-Central (Health Zones Kimpese, Kimpangu)	RT-PCR	11 (18.1)	2003–2012	[10]
Kinshasa (Binza-Meteo)	Rapid test NS1	111 (2.7)	2012	[16]
Mongala (Bumba), Kasai Oriental, Kinshasa (Kingabwa)	ELISA, PRNT	978 (3.8% in ELISA, of 32 ELISA (+) 9.4% (+) in PRNT) Serotypes: DENV-1, DENV-2, DENV-3	2013–2014	[9]
Kinshasa (Mbinza-Meteo)	Rapid test NS1, RT-PCR	96 (3.0% in rapid test NS1; 1.0% in RT-PCR)	2014	[30]

### Rift Valley fever

Rift Valley Fever virus has been reported in humans and cattle in DRC. A low rate (4%) of IgG antibodies from human against RVFV was reported in 1998 from Kisangani in the north-eastern region [11]. In 2009, Mulumba et al. [34] reported a seroprevalence of 20% among cattle in Katanga in the southern region. In 2018, a seroprevalence (IgG and IgM) of 2–16% among cattle was reported in three provinces (Nord-Kivu, Sud-Kivu, Ituri) from the eastern region [35]. In 2014, RVFV was also detected by RT-PCR from a pool of *Aedes* mosquitoes collected around Ndjili River in Kinshasa [13].

### Zika

Two studies on Zika were identified during the review. In the first study, of 453 archived sera collected country-wide from 2003–2011 that tested YFV negative were also negative for ZIKV virus using RT-PCR [10]. The second study analysed 978 blood samples collected during 2013–2014 showed that 34 samples (3.5%) were positive for Zika antibodies using ELISA. However, of the 34 samples that tested positive in ELISA, only one remained positive for ZIKV by PRNT [9]. This positive case of Zika was reported from the north-western part of DRC, from Sud-Ubangi Province bordering the Central African Republic.

### West Nile virus

In the DRC, antibodies against WNV in humans have been detected by virus neutralization tests, first in 1942 in Buta, Ango and Bondo from Bas-Uélé Province located in the north-eastern part, and later in 1963 from Gemena in Sud-Ubangi in the north-western part [36, 37]. The seroprevalence in these areas varied greatly from 2% to 45%. In 1998, a high seroprevalence of WVN (66%) was reported, during a febrile illness outbreak following heavy rainfall in Kisangani [11]. But no WNV antibodies or RNA were detected in samples from Kinshasa collected during 1999–2000 during a CHIKV outbreak [28]. The specific neutralizing antibodies against WNV in Haut-Uelé Province have been detected in chimpanzee in Ango, Bondo [32], in buffalo and elephant in the Garamba National Park [35]. In a survey carried out in Kinshasa in 2004, nine (30%) out of 20 horses were WNV seropositive using ELISA and western blot analysis [38]. Afterwards, a 12.5% seroprevalence of WNV was reported in Kinshasa [39]. These observations provide evidence of an animal role in the enzootic circulation of WNV in the DRC.

### O’nyong’nyong virus

O’nyong’nyong virus (ONNV) is a closer CHIKV related alphavirus vectored mainly by the *Anopheles* mosquito. Its RNA was detected by RT-PCR from a pool of *Anopheles* mosquito collected in Kinshasa [13]. Serological evidence of ONNV has been recorded in buffaloes from the Garamba National Park and duikers from the Ituri Rain Forest [33]. O’nyong’nyong outbreaks in humans were reported in neighbouring Uganda and Tanzania in 1996 [40]. However, no human case of ONNV infection has been reported in DRC.

### Unspecified *Flavivirus*, *Alphavirus* and *Bunyavirus*

Significantly high rates of neutralizing antibodies against flaviviruses and alphaviruses have been reported in buffaloes, elephants, gorillas and duikers from the Congo River basin [33]. In Kinshasa, alphavirus and flavivirus RNA in mosquito pools have been amplified with the primers targeting a conserved region of these genera, but none of these mosquito pools have been amplified with specific primers for CHIKV, ONNV, YFV, WNV or DENV [13]. In the Ango and Bondo territories from Bas-Uélé, up to 100% of antibodies against bunyavirus in humans have been reported [32]. All these observations are likely to indicate the presence of several unidentified mosquito-borne viruses circulating in DRC. Therefore, further studies are needed to extend investigations about other mosquito-borne virus species instead of focusing only on well-known MBVs.

### Mosquito vectors of arboviruses

Despite the medical and veterinary importance of mosquitoes, published reports on mosquito vectors of viruses are extremely rare in DRC. Entomological indices even during outbreaks within the regions with high mosquito-borne virus transmission risk are lacking in most cases. Moreover, *Ae. aegypti* and *Ae. simpsoni* were the most recorded in the regions where mosquito-borne virus outbreaks occurred. Yet, in certain areas, the presence of these *Aedes* species were not significantly recorded during outbreaks [41]. The entomological investigation during the first yellow fever outbreak at Matadi failed to collect a significant number of *Ae. aegypti* at that time [42]. Ten years later, *Ae. aegypti* predominated during the second yellow fever outbreak in Matadi and neighbouring townships [17], while in Gemena, *Ae. simpsoni* and *Ae. aegypti* were found in proportions of 90% and 10%, respectively during these outbreaks [18]. In the Bongo and Ango territories, where many mosquito-borne viruses reports are documented, *Ae. aegypti* predominated *Ae. simpsoni* [32]. In the four, last of the yellow fever outbreaks, *Ae. aegypti* larvae were collected from various breeding habitats in both natural and artificial breeding sites, while most positive breeding habitats were artificial [19–22]. A range of mosquitoes circulating in Kinshasa were found infected with different arboviruses [13]. O’nyong’nyong virus has been detected in *Anopheles* and unspecified flavivirus was detected in *Culex* mosquitoes (Table 3). *Aedes albopictus* has been recently established in Kinshasa [12], where the most recent outbreaks of chikungunya and dengue have been reported [16, 28, 30]; its implication in spreading mosquito-borne viruses in DRC needs to be investigated. It is important that vector species, distribution pattern, competence and virus infection rates are established. Furthermore, studies on the genetic population and vector competence will be more helpful in improving the understanding on transmission dynamics of mosquito-borne viruses in DRC.

### Risk factors of mosquito-borne viral disease occurrence

Numerous factors might have contributed to the emergence and re-emergence of mosquito-borne viral infections in DRC. These driving factors range from demographic, socioeconomic, environmental to climatic factors. Moreover, evidence of population movement as key driving factor of spreading yellow fever has been documented [41]. High population density seems to play a key role in the occurrence and spread of mosquito-borne virus outbreaks [11, 41]. For instance, the WNV outbreak in Kisangani started at army camps where immigrated soldiers from different locations across the whole country were concentrated before it spread to another area [11]. In addition, the increase of animal and human population movements is very likely to be considered as important drivers of mosquito-borne viral infections in DRC [11, 41]. The human exposures to forest activities and deforestation activities in cities have been associated to the yellow fever outbreaks [18]. In addition, significant rates of antibodies against YFV have been detected among chimpanzee from savannah areas compared to those captured in forest areas [32]. In Gemena city, the yellow fever outbreaks have been observed during large deforestation activities [18]. The overall geographical distribution of both mosquito-borne viruses and their potential vectors species seemed to be influenced by climate distribution pattern in DRC. The majority of outbreaks or positive cases occurred during the rainy seasons. The West Nile outbreak was preceded by heavy rainfall in Kisangani in 1998 [11]. Similarly, in Kinshasa, in 1999–2000, the large chikungunya outbreak followed heavy rainfall [28].

## Discussion

The overall findings indicate that mosquito-borne viral infections are an emerging and re-emerging health concern in the DRC. However, little attention has been paid and many questions on its epidemiology, socio-ecology and anthropology need to be addressed. While mosquito-borne viruses are re-emerging or emerging across the whole country, the north-western, north-eastern and western parts of the DRC are the most affected areas, experiencing multiple outbreaks compared to other parts of the country [9–11, 14, 15, 19–22]. This geographical distribution pattern of mosquito-borne viruses appeared to be linked to profile of the distribution, diversity and abundance of their potential vector species; most likely to be influenced by climate distribution pattern in DRC. In the eastern region, *Ae. aegypti* was rarely reported [43] compared to other regions [12, 44–48]. Indeed, the northern regions are experiencing an equatorial climate with rain throughout the year. From the central region to western region the climate is tropical with long rains, while in eastern and southern regions the climate is tropical but semi-arid. The Congo Basin has warmed between 0.2–0.3 °C per decade and reached 1 °C in 1992. The average annual precipitation has declined by 8–12% from 1960 to 1998. Climate change is manifested in DRC by short periods of heavy rainfall leading to multiple flooding events followed by early droughts [49, 50]. This considerable fluctuation in the climate regime causing either drought or flooding has an influence on vector abundance and diversity [51]. Most probably, heavy rainfalls are creating suitable mosquito breeding habitats, hence transmission of mosquito-borne viruses. Nevertheless, due to climate variability and increasing population movements, these mosquito species might establish successfully over time and change this general trend. Although, there is evidence of ZIKV, DENV and RVFV circulation in DRC, no epidemic has been reported so far.

This review indicates that there is a dearth of information as regards to vector and transmission indices of mosquito-borne viral infections in DRC. This underlines the importance of better identification of different local vectors in each region or specific area within regions, and to assess competence of the vectors to transmit mosquito-borne viruses. Intensive deforestation activities in Gemena city provided an opportunity for mosquito-borne viruses to emerge [18]. It is likely because of the degradation of the forest habitats during these activities, *Aedes* mosquitoes invaded the city and successfully established in their new habitats closer to humans, leading to a high human mosquito biting rate in a deforested area. Indeed, the increased carbon dioxide in the atmosphere due to deforestation warms up the climate and causes changes in rainfall patterns leading to conditions of flood or drought. The establishment of new suitable habitats is creating an opportunity for new mosquito species to proliferate [52]. This is in corroboration with observations from Brazil, where deforestation has been involved in sylvatic yellow fever outbreaks [53]. It was reported that mosquitoes which are known to be zoophilic and sylvatic have invaded the peridomestic and periurban settlements and started to feed on humans [53]. Similar observations from the Ivory Coast illustrated the impact of land-use modifications on the abundance, distribution, and host-seeking behaviour of *Aedes* mosquitoes [54]. A study in the Amazon concluded that deforestation is responsible for the increase in malaria incidence [55]. This observation could emphasize the impact of significant deforestation observed across the DRC in relation to the emergence of mosquito-borne viral diseases and repeated outbreaks in the country. There is currently a need to investigate the impact of environmental factors and the role of climate change on current mosquito-borne virus geographical distribution and predicting potential distribution in the future.

Climate change influences the frequency of mosquito-borne disease occurrence by affecting host and vector diversity, abundance, genetics and the pathogen infection load in the host and vector. Climate affects growth, survival and abundance of the vectors and virus replication load in the vector. Temperature is the major climate component susceptible to accelerate or decelerate mosquito reproduction and virus replication in the host depending on low and high temperature thresholds. Increased levels of precipitation lead to multiple microhabitats for oviposition and an efficient larval development pattern [51]. The higher rainfall levels due to climate change were associated with RFV outbreaks in eastern Africa during 2015–2016 [56].

RVFV is remarkably distributed in the eastern region of the DRC starting from the South to the North extremity. This region constitutes long borders with Zambia, Tanzania, Burundi, Rwanda, Uganda and South Sudan. Most of these countries are known to be endemic of Rift Valley fever [56–60]. It is likely that the active and uncontrolled migration of human and animal population might have played a key role in spreading of RVFV in the DRC. Considering the largest cattle population in eastern DRC, the situation represents a real health threat that needs more attention. The role of population movement in the emergence and spread of mosquito-borne viruses have been illustrated in a recent study of a yellow fever outbreak which started in Angola and was imported to the DRC through trans-boundary trade between the two countries [41]. Environmental and climate factors, human activities and population movement are likely contributing enormously to the emergence and re-emergence of mosquito-borne viruses. An exponential demographic growth of Kinshasa with poor environmental hygiene and low urbanisation level is likely to have favoured *Aedes* mosquito productivity and might explain in part the frequent chikungunya and yellow fever outbreaks in the city.

## Conclusions

Despite the limited information on mosquito-borne viruses and their vectors in DRC, available evidence indicates the wide-range of mosquito-borne virus species, potential hosts and vector species occurring in the country. The diversity of mosquito-borne viruses is underestimated, as well the current mosquito-borne viral disease burden and its driving factors. The tendency is that the continuous geographical expansion of mosquito-borne viruses leads to fears that multiple epidemic outbreaks are likely to arise in the DRC. There is a dearth of information on mosquitoes, in particular *Aedes*, which is known to be the main vector of major mosquito-borne viruses. The lack of information is a barrier to appropriate knowledge on the ecology and epidemiology of mosquito-borne viruses, which is crucial in the design of appropriate interventions. Evidence for circulation of mosquito-borne viruses in livestock and domestic animals from the DRC, and the increasing mosquito-borne virus distribution over time and space, call for robust holistic mosquito-borne virus surveillance systems, including One Health approaches with emphasis on integrated vector control.

## Data Availability

The datasets supporting the conclusions of this article are included within the article.

## Abbreviations

CHIKV: chikungunya virus
DENV: dengue virus
RVFV: Rift Valley fever virus
YFV: yellow fever virus
WNV: West Nile virus
ZIKV: Zika virus
DRC: Democratic Republic of the Congo
ELISA: enzyme‐linked immunosorbent assay
PRNT: plaque reduction neutralization test
RT-PCR: reverse transcription polymerase chain reaction
